# Consistent condom utilization among sexually active HIV positive individuals in Sub-Saharan Africa: systematic review and meta-analysis

**DOI:** 10.1038/s41598-024-56574-5

**Published:** 2024-03-10

**Authors:** Destaw Endeshaw, Getenet Dessie, Ayele Semachew Kasa, Aklilu Endalamaw, Tadesse Dagget Tesfaye, Shiferaw Birhanu, Teshager Woldegiyorgis Abate, Sahileslassie Afewerk, Eyob Ketema Bogale, Yinager Workineh

**Affiliations:** 1 College of Medicine and Health Sciences, Bahir Dar University, Bahir Dar, Ethiopia; 2 Centre for Epidemiology and Population Health, The Australian National University, Canberra, Australia; 3 School of Public Health, The University of Queensland, Brisbane, Australia; 4 Faculty of Nursing, University of Alberta Edmonton, Edmonton Clinic Health Academy, Edmonton, AB T6G 1C9 Canada

**Keywords:** Prevalence, Consistent condom utilization, Sub-Saharan Africa, Disease prevention, HIV infections, Epidemiology

## Abstract

This study aimed to ascertain the pooled prevalence and trend of consistent condom use in Sub-Saharan Africa, addressing the fragmented and inconsistent research on its role in preventing HIV transmission. In this meta-analysis, we systematically searched electronic databases such as PubMed, Embase, Scopus, Web of Science, Global Index Medicus, ScienceDirect, Africa-Wide Information (via EBSCOhost), as well as clinical trial registries, and the search engine Google Scholar. All necessary data were extracted using a standardized data extraction format. The data were analyzed using STATA 17 statistical software. Heterogeneity among the studies was assessed using the *I*^2^ test. A random-effect model was computed to estimate the pooled rate of consistent condom utilization. This meta-analysis, which included thirty-three full-text studies, found a pooled prevalence of 44.66% (95% CI 18.49–70.83; *I*^2^ = 0.00%) for consistent condom use in Sub-Saharan Africa. While the prevalence fluctuated between 2007 and 2022, the year-to-year variations were not statistically significant. The current study identified low rates of consistent condom use, with utilization fluctuating annually in the study area. Therefore, uncovering the underlying reasons and addressing barriers to consistent condom use is crucial in the region.

## Introduction

Great efforts have been made to end the Human Immunodeficiency Virus/Acquired Immunodeficiency Syndrome (HIV/AIDS) epidemic by 2030, yet it remains a global problem in post Millennium Development Goal (MDG) era. In 2022, approximately 39.0 million people were living with HIV globally. Among these, two-thirds (25.6 million) were in Africa^1^. Among the 39.0 million people living with HIV globally, 20.6 million were women, and 1.5 million were children. Additionally, 630,000 people died from AIDS-related illnesses in 2022^2^.

Modeled estimates show that new infections declined from a peak of 3.4 million in 1996 to 1.8 million in 2017. However, this progress was slower than what was necessary to meet the 2020 milestone of fewer than 500,000 new infections^3,4^, indicating more effort is required.

Condoms are central to a combination HIV prevention approach and serve as cost-effective tools for preventing other sexually transmitted infections and unintended pregnancies^5^. Preventing unintended pregnancy among HIV-positive women constitutes a critical and cost-effective approach to the primary prevention of mother-to-child (PMTCT) of HIV. It is also a global public health priority to address the compromised state of maternal and child health in areas with high HIV prevalence^6^.

Despite a decrease in new HIV infections and AIDS-related deaths in recent years^7^, the international goal of reducing sexual transmission among youth and adults by 50% from 2010 to 2015 was not met^8^. This failure to achieve the target is associated with inconsistent condom use, attributed to limited knowledge about HIV and the belief that using condoms reduces sexual pleasure^9^.

Although consistent condom utilization (CCU) is vital for the prevention of HIV transmission, the studies conducted on this issue were fragmented and inconsistent in Sub-Saharan Africa (SSA). The prevalence of CCU ranges from 16.1%^10^ to 78.9%^11^ which showed a great variation across different geographical settings and different periods. Hence, evidence related to CCU should be identified by researchers to provide the leadership that will enable 90–90–90 to succeed in the globe in general and in SSA in particular. Therefore, the current study aimed to assess the pooled prevalence and trend of CCU among sexually active HIV positive individuals in SSA countries, 2023.

## Methods

### Protocol and registration

The findings presented in this review adhere to the guidelines outlined in the Preferred Reporting Items for Systematic Review and Meta-Analysis (PRISMA) statement^12^ (Supplementary Table S1). The protocol for this review has been prospectively registered in the International Prospective Register of Systematic Reviews (PROSPERO), under the registration number CRD42022382283.

### Search strategy and selection criteria

To gather relevant studies, we searched through various databases, including PubMed, Embase, Scopus, Web of Science, Global Index Medicus, ScienceDirect, Africa-Wide Information (via EBSCOhost), and clinical trial registries such as ClinicalTrials.gov and the International Clinical Trials Registry Platform (ICTRP). We also utilized Google Scholar and accessed the online libraries of Addis Ababa University and Bahir Dar University to further explore literature. Additionally, we manually reviewed the reference lists of included articles to identify any additional relevant studies.

The core search terms and phrases included "prevalence," "consistent," "utilization," "condom," "HIV," "positive," "person," "individual," and the names of sub-Saharan African countries. We used various techniques in our search strategies, such as truncation (*), Boolean operators (OR and AND), and phrase searching ("…"). Furthermore, we included MeSH terms in PubMed, Emtree terms in Embase, and synonyms to ensure comprehensive searches. The detailed search strategy is presented in Table (Supplementary Table S2).

### Inclusion and exclusion criteria

Our study included all types of studies reported in the English language on the prevalence of consistent condom utilization, without restrictions on the study period. We considered articles available in our search sources from December 20, 2022, to January 30, 2023. We excluded articles without abstracts or full texts, anonymous reports, editorials, and qualitative studies.

### Quality assessment and data abstraction procedure

The retrieved studies were imported into Endnote version 7, a reference manager software, to eliminate duplicate studies. Four authors (YW, TW, DE, and SB) independently reviewed and screened the titles and abstracts of the identified studies. Any disagreements that arose were resolved based on pre-established article selection criteria. To assess the quality of each study, we utilized The Newcastle–Ottawa Scale^13^, which was adapted for the systematic review to evaluate cross-sectional studies^14^. The assessment tool consists of three domains: selection, comparability, and outcome, each encompassing specific items detailed in the supplementary file (Supplementary Table S3). Each original study underwent independent evaluation by the four authors using this tool. In case of disagreements among the authors, a consensus was reached by averaging the scores provided by the four authors. The inter-rater agreement was calculated using a Fleiss kappa statistic that yielded a value of 0.226 (95% CI 0.222–0.231, *p* value < 0.001), indicating a fair level of agreement (Supplementary Table S4).

### Outcome measurement

Consistent condom utilization is the percentage of respondents who used a condom every time they had sex with any non-spouse or non-cohabiting partner over time^15^. The prevalence was determined by dividing the total number of HIV-positive individuals who consistently used condoms by the total number of HIV-positive individuals included in the study, then multiplying by 100.

### Data extraction and analysis

For data extraction, a standardized format adapted from the data extraction format of the Joanna Briggs Institute (JBI)^16^ was utilized. Four authors independently extracted the necessary data using this format. In case of any discrepancies during the data extraction process, they were resolved through discussion and consensus. The data extraction format consisted of the primary author’s name, publication year, study site, study design, response rate, sample size, participant gender, and prevalence of consistent condom use with a 95% confidence interval (CI).

STATA version 17 statistical software was used for meta-analysis. Pooled analysis was conducted using a random-effects model^17^. The level of heterogeneity among the studies was assessed using the I-squared statistic, with values of 25%, 50%, and 75% indicating low, moderate, and high heterogeneity, respectively^17,18^. However, in this analysis, there was no heterogeneity between studies (*I*^*2*^ = 0.00%). Sub-group analysis was done based on study region, design, and participants’ gender. To examine publication bias, we utilized funnel plots and performed Begg's and Egger's regression tests^19^ for a more objective assessment. Trim and fill analyses were also performed. Furthermore, sensitivity analysis was employed to assess the influence of individual studies on the overall estimation.

## Results

### Search results

**Figure 1:**
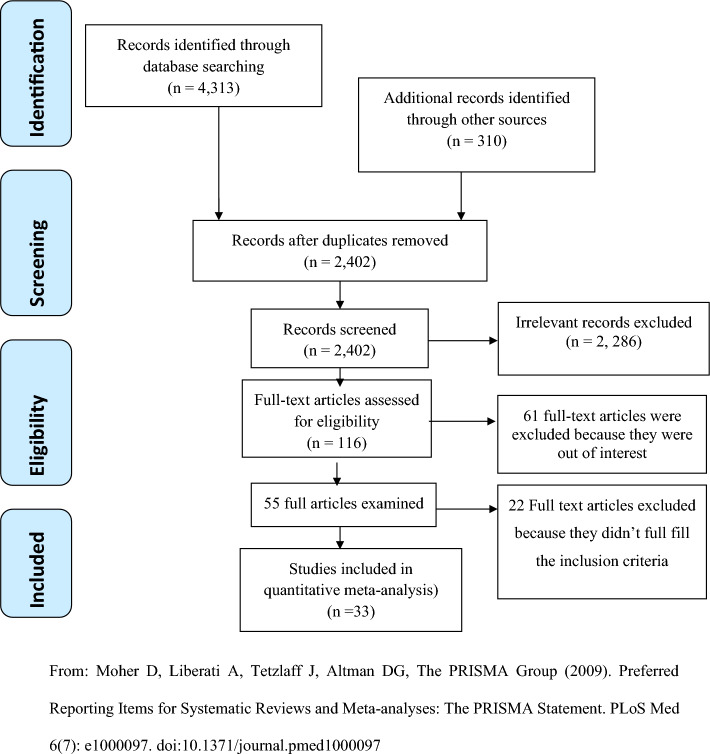
Flowchart of a selection of studies for a systematic review and meta-analysis of the prevalence of CCU in Sub-Saharan Africa, 2023.

The initial search yielded 4,623 articles from various sources. After removing duplicates, 2402 unique articles were identified. Among these, 2,286 irrelevant records were excluded, leaving 116 articles for further review. Out of these, 61 articles were excluded after reviewing their titles and abstracts due to lack of relevance. Subsequently, 55 full-text articles were thoroughly assessed based on the inclusion criteria^10,11,20–72^. Finally, a total of 33 studies^10,11,20–22,24–27,29,32,34,36,38,45–55,65–72^ meeting the inclusion criteria were included in the meta-analysis (Fig. 1).

### Characteristics of reviewed studies

This review included thirty-three studies published between 2007 and 2022, with a cumulative sample size of 11,499. The majority of the included studies originated from Ethiopia (eleven) and Nigeria (ten). Thirty-one studies employed a cross-sectional study design, while two utilized a cohort study design (Table 1).

**Table 1:** Descriptive summary of 33 studies included in the meta-analysis of the prevalence of consistent condom utilization in Sub-Saharan African countries, 2023.

Authors name	Country	Region	Study design	Response rate (%)	Sample size	Number of people with outcome	Participant Gender	Prevalence of CCU (%)
Ezeala-A et al. (2017)	Nigeria	West Africa	cross-sectional	100	207	113	Both	61.40
Ayiga A. et al. (2012)	Uganda	East Africa	cross-sectional	100	269	175	Both	65
Shewamene Z. et al. (2015)	Ethiopia	East Africa	cohort	100	317	250	Both	78.9
Nduka I. et al. (2014)	Nigeria	West Africa	cross-sectional	100	248	73	Female	29.4
Conserve D. et al. (2012)	Tanzania	East Africa	cross-sectional	100	731	118	Both	16.1
Macharia A et al. (2015)	Kenya	East Africa	cross-sectional	100	422	242	Female	57.4
Addis K. et al. (2014)	Ethiopia	East Africa	cross-sectional	98	351	199	Female	56.7
Ali M. et al. (2019)	Ethiopia	East Africa	cross-sectional	90	358	130	Both	55.8
Ayoola D. et al. (2014)	Nigeria	West Africa	cross-sectional	100	302	147	Both	48.8
Yalew E. et al. (2012)	Ethiopia	East Africa	cross-sectional	100	222	111	Both	50
Busari M. et al. (2019)	Nigeria	West Africa	cross-sectional	100	212	92	Both	43.4
Salaudeen et al. (2014)	Nigeria	West Africa	cross-sectional	100	231	119	Both	51.5
Ezeanochi M. et al. (2009)	Nigeria	West Africa	cross-sectional	100	55	23	Female	48.9
Haddad B. et al. (2015)	Malawi	South Africa	cohort	100	200	71	Female	35.9
Pilapil M. et al. (2016)	Cameroon	West Africa	cross-sectional	100	84	23	Female	27
Haddad B. et al. (2018)	Malawi	South Africa	cross-sectional	100	474	210	Both	40.3
Berhane Y. et al. (2015)	Ethiopia	East africa	cossectional	100	201	112	Female	55.7
Dessie Y. et al. (2011)	Ethiopia	East africa	cossectional	100	601	379	Both	63.1
Yeshaneh A. et al. (2021)	Ethiopia	East Africa	cross-sectional	100	419	206	Both	49.2
Dereje L. et al. (2021)	Ethiopia	East Africa	cross-sectional	91	535	288	Both	59.1
Biruk. et al. (2020)	Ethiopia	East Africa	cross-sectional	92	391	184	Both	51.4
Rahel. et al. (2020)	Ethiopia	East Africa	cross-sectional	100	677	306	Both	45.2
Adefala et. al (2020)	Nigeria	West Africa	cross-sectional	90.5	315	131	Both	46
Tadesse and Gelagay (2019)	Ethiopia	East Africa	cross-sectional	100	317	123	Both	38.8
Ilesanmi et al. (2014)	Nigeria	West Africa	cross-sectional	100	427	324	Both	75.9
Aboubacrine et al. (2007)	Burkina Faso and Mali	West Africa	cross-sectional	100	212	80	Both	38
Ajayi et al. (2022)	Nigeria	West Africa	cross-sectional	100	360	142	Female	39.4
Ankunda et al. (2016)	Uganda	East Africa	cross-sectional	100	69	32	Both	46.4
Keetile et al. (2018)	Botswana	South Africa	cross-sectional	100	1065	938	Both	88.1
Nakiganda et al. (2017)	Uganda	East Africa	cross-sectional	100	517	98	Both	19
Obi et al. (2009)	Nigeria	West Africa	cross-sectional	100	104	29	Both	27.9
Ragnarsson et al. (2011)	Kenya	East Africa	cross-sectional	100	368	264	Both	71.7
Wagner et al. (2010)	Uganda	East Africa	cross-sectional	100	258	88	Both	34.1

### Meta-analysis

#### Risk bias Assessment

The studies included in this systematic review and meta-analysis had no considerable risk based on the Newcastle–Ottawa Scale quality appraisal criteria. Therefore, all the studies were considered in this review^10,11,20–22,24–27,29,32,34,36,38,45–55,65–72^ (Table 1).

#### Publication bias

**Figure 2:**
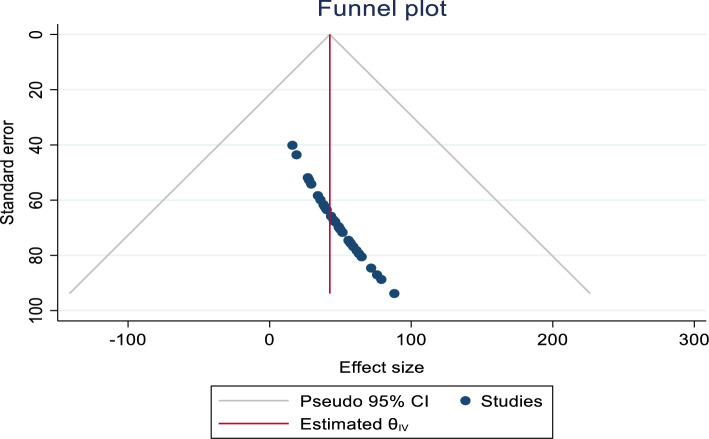
Funnel plot For publication bias, Logprop or Lnp (log of proportion) represented in the X-axis and standard error of log proportion in the Y-axis.

**Figure 3:**
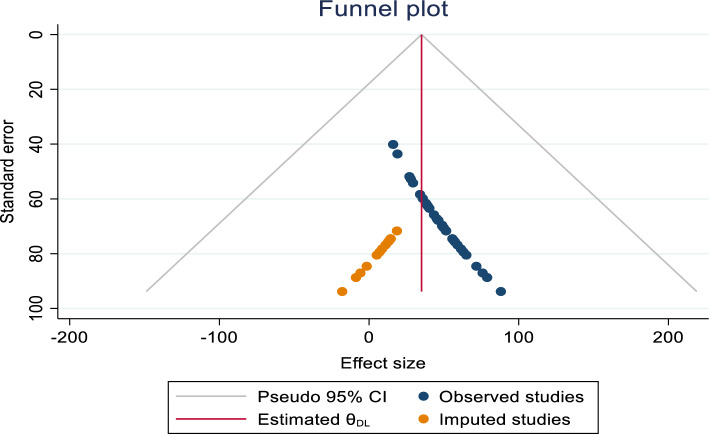
The trim fill analysis showed the pooled prevalence when the unpublished studies were filled.

A funnel plot indicated an asymmetrical distribution (Fig. 2), and Begg’s test yielded statistically significant results (*p* < 0.001). However, Egger’s test showed insignificant results for estimating the prevalence of CCU. To assess the impact of publication bias on the pooled analysis, trim fill analysis was conducted, and thirteen studies were filled. In this analysis, the pooled prevalence of CCU became 35.1% (CI 15.3%, 54.8%). Consequently, the confidence interval suggests no significant difference in the pooled prevalence of CCU (Fig. 3).

#### Sensitivity analysis

**Figure 4:**
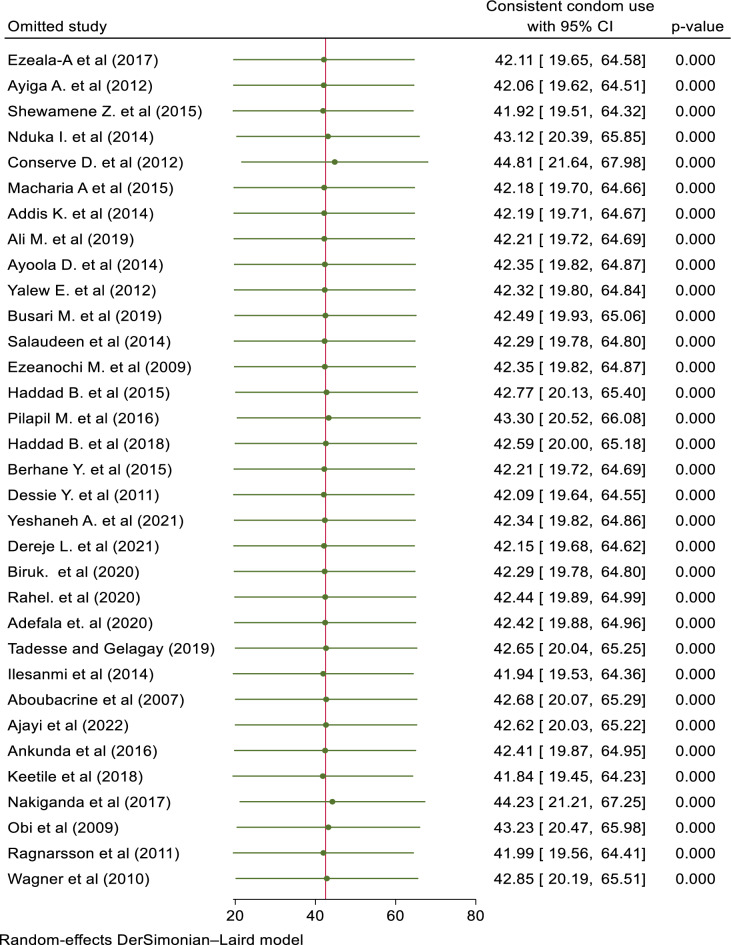
The sensitivity analysis showed the pooled prevalence when the studies omitted step-by step.

As shown in the figure, no individual study had an impact on the overall estimation of the result in the leave-one-out meta-analysis (Fig. 4).

#### Prevalence of consistent condom utilization

**Figure 5:**
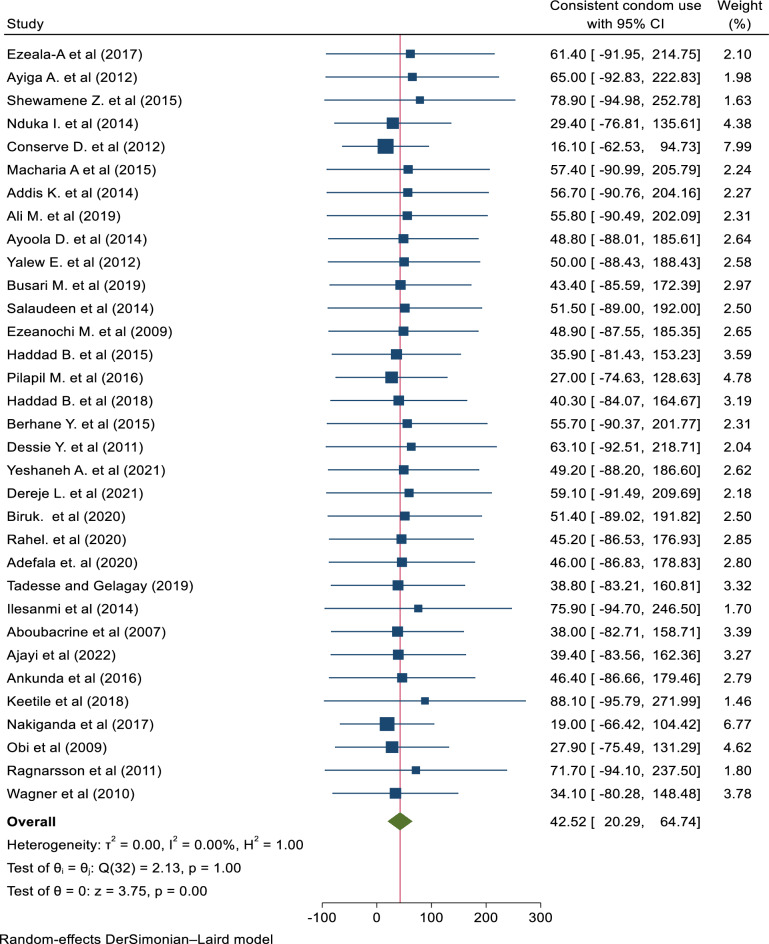
A forest plot that describes the meta-analysis result of consistent condom utilization among sexually active HIV-positive individuals in Sub-Saharan Africa, 2023 (n = 33).

In this review, the prevalence of consistent condom utilization ranged from 16.1% to 78.9%^10,11^. Using the random effects meta-analysis model, the pooled prevalence of CCU was 42.52% (95% CI; 20.29, 64.74; *I*^2^ = 0.00%). The estimated overall prevalence of CCU is presented in a forest plot (Fig. 5).

#### Subgroup analysis result of consistent condom use across sub-Sharan Africa

**Figure 6:**
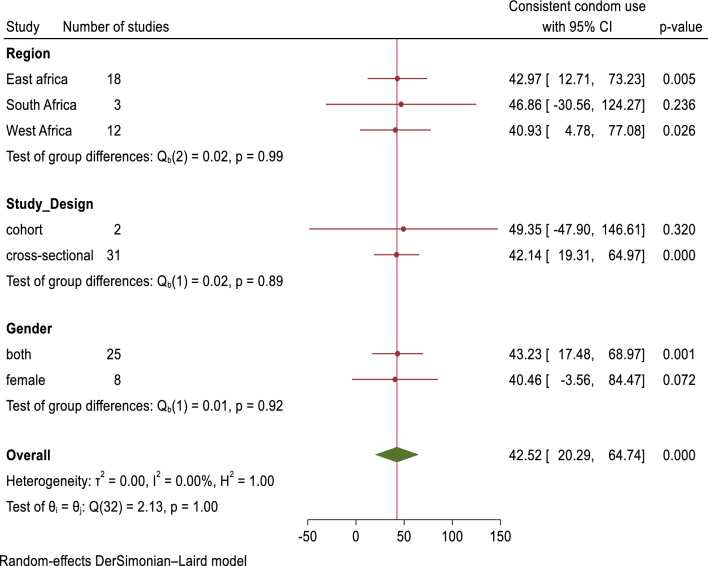
A forest plot that describes the sub-group meta-analysis result of consistent condom utilization among sexually active HIV-positive individuals in Sub-Saharan Africa, 2023 (n = 33).

We have investigated whether study region, study design, sample size, publication year, and participants’ gender influence variability in the pooled estimation, utilizing a meta-regression model. However, none of these factors showed statistical significance (Supplementary Table S5). Subgroup analysis was done based on region, study design, and participants’ gender. This analysis revealed that consistent condom use was relatively higher in the Southern Africa region, among studies employing a cohort design, and in studies focusing exclusively on women (Fig. 6).

#### Trend of CCU

**Figure 7:**
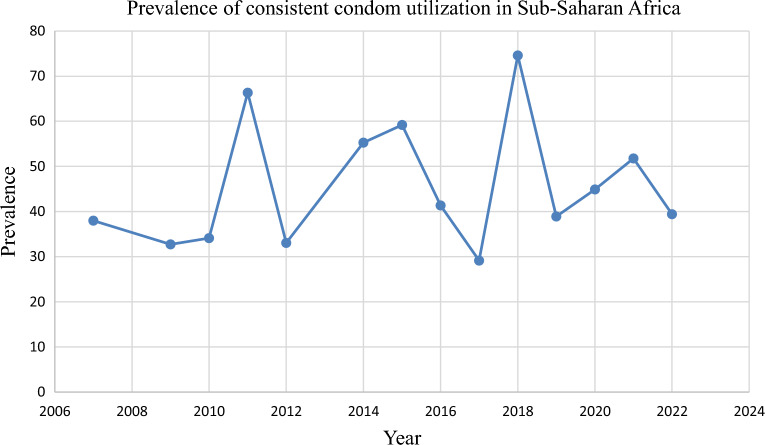
Trend of consistent condom utilization among HIV-positive individuals in Sub-Saharan African countries, 2023.

The prevalence of consistent condom use fluctuated between 2007 and 2022 (Fig. 7). However, our meta-regression analysis indicated that the year-to-year variability was not statistically significant (Supplementary Table S5).

## Discussion

This systematic review and meta-analysis investigate consistent condom use among sexually active HIV-positive individuals in Sub-Saharan Africa. Globally, consistent condom use rates range from 4 to 52.4% among young, sexually active individuals^73,74^. In Sub-Saharan Africa, HIV transmission still constitutes a public health concern, with many adolescents and young adults engaging in unprotected sex and transactional sex^73,75^. This systematic review and meta-analysis finding also indicated that consistent condom utilization among sexually active HIV-positive individuals in SSA is low (42.5%). This finding is lower than a study conducted in the Asia–Pacific region (54–57%)^44^. Even if the response to HIV was started four decades before, CCU is low in SSA due to the existence of different harmful traditional practices such as age restrictions, gender norms, religious norms, stigma, and insufficient supply and, in some places, laws that make it an offense to carry condoms^76,77^. Many countries in Sub-Saharan countries also prohibit condom promotion and distribution in schools and other venues where adolescents socialize^76^. Hence, this brings a low level of consistent condom utilization in SSA countries as compared with Asian Pacific countries. In the current study, there is no substantial heterogeneity of consistent condom utilization across SSA.

The prevalence of consistent condom utilization was swinging up and down from 2007 to 2022. However, year-to-year variability was not statistically significant. Consistent condom utilization by HIV-positive individuals varies from year to year. This could be because, in recent years there is decrement in international funding for condom procurement in Sub-Saharan Africa. Moreover, domestic funding has not sufficiently increased its focus on the issue. Finally, this leads to stalled condom promotion and demand creation due to a lack of funding and decreased investment in several Sub-Saharan countries^78^.

Currently, the AIDS response and the broader global community have united around the goal of achieving 90–90–90 and ending the AIDS epidemic as a public health threat^79^. However, the low level of consistent condom utilization and its variability from year to year among HIV-positive individuals in Sub-Saharan countries will threaten the achievement of the 90–90–90 approach by 2030. Hence, focused efforts by health professionals should be given to developing skills that allow consistent condom utilization and overcome the specific obstacles that reduce the efficient and effective condom use in Sub-Saharan Africa's HIV-positive populations.

The low consistent condom utilization among sexually active HIV-positive individuals in Sub-Saharan Africa highlights an urgent need for targeted interventions to promote safer sexual practices and reduce HIV transmission rates within this vulnerable population.

## Limitation

Limiting studies to publications in English may introduce bias to the overall findings in sub-Saharan Africa.

## Conclusion

There is low consistent condom utilization among sexually active HIV-positive individuals in SSA. Additionally, consistent condom use has fluctuated over the years in the region. Therefore, it is crucial to investigate the underlying reasons and address the barriers. This endeavor can enhance HIV prevention efforts and, ultimately, improve public health outcomes.

### Supplementary Information

Supplementary Information.

## Data Availability

All data generated or analyzed during this study are included in the manuscript or supplementary information.

## Abbreviations

AIDS: Acquired immuno-deficiency syndromes
CI: Confidence interval
CCU: Consistent condom utilization
HIV: Human immuno virus
SSA: Sub-Saharan Africa
WHO: World Health Organization
