# Supplementary material for: Consistent condom utilization among sexually active HIV positive individuals in Sub-Saharan Africa: systematic review and meta-analysis
Source: Sci Rep. 2024 Mar 10;14:5837. doi: 10.1038/s41598-024-56574-5 (PMC10925590; doi:10.1038/s41598-024-56574-5)
Supplement: Supplementary file 1 — Supplementary Information. [file 41598_2024_56574_MOESM1_ESM.pdf]

Consistent condom utilization among sexually active HIV positive individuals in Sub-Saharan Africa: Systematic review and meta-analysis

Destaw Endeshaw[1], Getenet Dessie[1,2], Ayele Semachew Kasa[1], Aklilu Endalamaw[1,3], Tadesse Dagget Tesfaye[1], Shiferaw Birhanu[1], Teshager Woldegiyorgis Abate[1,4], Sahileslassie Afewerk[1], Eyob Ketema Bogale[1], Yinager Workineh[1]

1 College of Medicine and Health Science, Bahir Dar University, Bahir Dar, Ethiopia.

2 The Australian National University, Center for Epidemiology and Population Health

3 School of Public Health, the University of Queensland, Brisbane, Australia

4 Faculty of Nursing, University of Alberta Edmonton, Edmonton Clinic Health Academy, Edmonton AB T6G 1C9, Canada

Supplementary Table S1: PRISMA 2009 Checklist

Section/topic	#	Checklist item	Reported on page #
**TITLE**			
Title	1	Identify the report as a systematic review, meta-analysis, or both.	1
**ABSTRACT**			
Structured summary	2	Provide a structured summary including, as applicable: background; objectives; data sources; study eligibility criteria, participants, and interventions; study appraisal and synthesis methods; results; limitations; conclusions and implications of key findings; systematic review registration number.	2
**INTRODUCTION**			
Rationale	3	Describe the rationale for the review in the context of what is already known.	3-4
Objectives	4	Provide an explicit statement of questions being addressed with reference to participants, interventions, comparisons, outcomes, and study design (PICOS).	4
**METHODS**			
Protocol and registration	5	Indicate if a review protocol exists, if and where it can be accessed (e.g., Web address), and, if available, provide registration information including registration number.	4
Eligibility criteria	6	Specify study characteristics (e.g., PICOS, length of follow-up) and report characteristics (e.g., years considered, language, publication status) used as criteria for eligibility, giving rationale.	5
Information sources	7	Describe all information sources (e.g., databases with dates of coverage, contact with study authors to identify additional studies) in the search and date last searched.	4
Search	8	Present full electronic search strategy for at least one database, including any limits used, such that it could be repeated.	4-5
Study selection	9	State the process for selecting studies (i.e., screening, eligibility, included in systematic review, and, if applicable, included in the meta-analysis).	5-6
Data collection process	10	Describe method of data extraction from reports (e.g., piloted forms, independently, in duplicate) and any processes for obtaining and confirming data from investigators.	6
Data items	11	List and define all variables for which data were sought (e.g., PICOS, funding sources) and any assumptions and simplifications made.	6
Risk of bias in individual studies	12	Describe methods used for assessing risk of bias of individual studies (including specification of whether this was done at the study or outcome level), and how this information is to be used in any data synthesis.	5-6
Summary measures	13	State the principal summary measures (e.g., risk ratio, difference in means).	6
Synthesis of results	14	Describe the methods of handling data and combining results of studies, if done, including measures of consistency (e.g., I^2) for each meta-analysis.	6

Section/topic	#	Checklist item	Reported on page #
Risk of bias across studies	15	Specify any assessment of risk of bias that may affect the cumulative evidence (e.g., publication bias, selective reporting within studies).	7
Additional analyses	16	Describe methods of additional analyses (e.g., sensitivity or subgroup analyses, meta-regression), if done, indicating which were pre-specified.	7
**RESULTS**			
Study selection	17	Give numbers of studies screened, assessed for eligibility, and included in the review, with reasons for exclusions at each stage, ideally with a flow diagram.	7
Study characteristics	18	For each study, present characteristics for which data were extracted (e.g., study size, PICOS, follow-up period) and provide the citations.	7-10
Risk of bias within studies	19	Present data on risk of bias of each study and, if available, any outcome level assessment (see item 12).	11
Results of individual studies	20	For all outcomes considered (benefits or harms), present, for each study: (a) simple summary data for each intervention group (b) effect estimates and confidence intervals, ideally with a forest plot.	11
Synthesis of results	21	Present results of each meta-analysis done, including confidence intervals and measures of consistency.	11
Risk of bias across studies	22	Present results of any assessment of risk of bias across studies (see Item 15).	11
Additional analysis	23	Give results of additional analyses, if done (e.g., sensitivity or subgroup analyses, meta-regression [see Item 16]).	11-12
**DISCUSSION**			
Summary of evidence	24	Summarize the main findings including the strength of evidence for each main outcome; consider their relevance to key groups (e.g., healthcare providers, users, and policy makers).	12-13
Limitations	25	Discuss limitations at study and outcome level (e.g., risk of bias), and at review-level (e.g., incomplete retrieval of identified research, reporting bias).	14
Conclusions	26	Provide a general interpretation of the results in the context of other evidence, and implications for future research.	14
**FUNDING**			
Funding	27	Describe sources of funding for the systematic review and other support (e.g., supply of data); role of funders for the systematic review.	NA

From: Moher D, Liberati A, Tetzlaff J, Altman DG, The PRISMA Group (2009). Preferred Reporting Items for Systematic Reviews and Meta-Analyses: The PRISMA Statement. PLoS Med 6(7): e1000097. doi:10.1371/journal.pmed1000097

For more information, visit: **www.prisma-statement.org**.

Supplementary Table S2: Search strategy

Sources	Combined final search terms	Number of studies identified
PubMed	(((condoms[MeSH Terms]) AND ((use[Text Word]) OR (utilization[All Fields]))) AND ((HIV infected[All Fields]) OR (HIV positive[All Fields]))) AND (Africa South of the Sahara[MeSH Terms])	485
Global Index Medicus	tw:(("condom use" OR "condom utilization") AND ("HIV infected" OR "HIV positive" OR art)) AND (la:("en"))	49
Embase	'condom'/exp AND ('use':ti ORab ORkw ORde ORdn ORdf ORmn ORtn OR 'utilization') AND ('hiv infected' OR 'hiv positive') AND 'africa south of the sahara'/exp AND ([embase]/lim OR [embase classic]/lim OR [preprint]/lim) AND [english]/lim	257
Scopus	(TITLE-ABS-KEY ("Consistent condom use" OR "condom use" OR "condom utilization" OR "Consistent condom utilization" OR condom*) AND TITLE-ABS-KEY ("HIV infected" OR "HIV Positive" OR "ART user*" OR art OR "patients with hiv" OR "patients receiving ART" OR "patients with human immuno-deficiency virus") AND TITLE-ABS-KEY (Sub-Saharan Africa#)) AND (LIMIT-TO (LANGUAGE , "English"))	44
Web of Science	Consistent condom utilization (All Fields) AND sexually active (All Fields) AND HIV positive individuals (All Fields) AND Sub-Saharan Africa# (All Fields)<br>Limit to English (Languages)	2121
Africa-Wide Information (EBSCOhost)	(TI condom use OR TI condom utilization) AND (TI HIV infected OR TI HIV positive OR TI ART)	50
ScienceDirect	Consistent condom utilization among sexually active HIV positive individuals (filters applied)	1242
ClinicalTrials.gov	Condom Utilization	9
International Clinical Trials Registry Platform (ICTRP)	Condom Use	56
Google Scholar	allintitle: condom use HIV positive condom "condom use"	102
Other (Manual & repositories)		208
**Total**		**4623**

Instead of Sub-sahran africa#, the names of the countries listed below have been entered separately.
#= (Angola OR Benin OR Botswana OR Burkina Faso OR Burundi OR Cameroon OR Cape Verde OR Central African Republic OR Chad OR Comoros OR Republic of the Congo OR Democratic Republic of the Congo OR Cote d'Ivoire OR Djibouti OR Equatorial Guinea OR Eritrea OR Ethiopia OR Gabon OR The Gambia OR Ghana OR Guinea OR Guinea-Bissau OR Kenya OR Liberia OR Madagascar OR Malawi OR Mali OR Mauritania OR Mauritius OR Mozambique OR Namibia OR Niger OR Nigeria OR Rwanda OR Sao Tome and Principe OR Senegal OR Seychelles OR Sierra Leone OR Somalia OR South Africa OR South Sudan OR Sudan OR Swaziland OR Tanzania OR Togo OR Uganda OR Zambia OR Zimbabwe)

Supplementary Table S3: Scoring of the quality of articles by authors using The Newcastle-Ottawa Quality Assessment tool, 2023.

		Selection				Comparability		Outcome		
Study	Quality assessor	Representativeness of sample (*, *)	Sample size (*)	Non-respondents (*)	Ascertainment (**)	Study controls for most important factor (*)	The study control for any additional factor (*)	Assessment of the outcome (*)	Statistical test (*)	Total score
Ezeala-Adikaibe. BA et al, 2017	YW	1	1	1	1	1	1	1	1	7
	AE	1	1	1	1	1	1	1	1	
	GD	1	0	0	1	1	1	1	0	
	TW	1	1	1	1	1	0	1	1	
Ayiga A. et al, 2012	YW	1	0	0	2	1	1	1	0	6
	AE	0	1	1	1	1	1	1	1	
	GD	1	0	1	1	1	1	1	0	
	TW	0	1	1	0	1	1	1	0	
Shewamene Z. et al, 2015	YW	1	1	1	0	1	0	0	1	6
	AE	0	1	1	1	1	0	0	1	
	GD	1	1	1	1	1	1	1	0	
	TW	1	0	1	2	1	0	0	1	
Nduka I. et al, 2014	YW	1	1	1	1	1	1	1	1	7
	AE	1	1	1	1	1	1	1	0	
	GD	1	0	0	1	1	1	1	1	
	TW	1	1	1	1	1	0	1	1	
Conserve D. et al, 2012	YW	2	0	1	2	1	1	1	0	8
	AE	2	1	1	2	1	0	1	1	
	GD	2	1	1	1	1	1	1	0	
	TW	1	1	1	0	1	1	1	1	
Macharia A et al, 2015	YW	2	0	0	2	1	1	1	0	8
	AE	2	1	1	2	1	0	1	1	
	GD	2	1	1	1	1	1	1	0	
	TW	1	1	1	2	1	1	1	1	
Addis K. et al, 2014	YW	1	1	1	1	1	0	1	1	8
	AE	1	1	1	1	1	1	1	1	
	GD	1	1	1	2	1	1	1	1	
	TW	1	1	1	2	1	1	0	1	
Ali M. et al, 2019	YW	1	1	1	1	1	1	1	1	7
	AE	1	1	1	1	1	1	1	1	
	GD	1	1	1	1	1	1	1	0	
	TW	1	1	1	1	1	0	1	1	
Ayoola D. et al, 2014	YW	1	1	1	1	1	0	1	1	8
	AE	2	1	1	1	1	1	1	0	
	GD	2	1	1	2	1	1	0	1	
	TW	1	1	1	2	1	1	0	1	
Yalew E. et al, 2012	YW	1	1	1	1	1	1	1	1	7
	AE	2	1	1	2	1	1	1	0	
	GD	1	1	0	1	1	1	0	1	
	TW	2	1	1	1	1	1	1	1	
	YW	1	0	1	0	1	1	1	1	6

Busari M. et al, 2019	AE	1	1	1	1	1	0	1	0	
	GD	1	0	1	1	0	1	1	1	
	TW	0	1	1	1	1	1	1	0	
Salaudeen et al, 2014	YW	1	1	1	1	1	1	1	1	7
	AE	1	1	1	1	1	1	1	1	
	GD	1	0	0	1	1	1	1	0	
	TW	1	1	1	1	1	0	1	1	
Ezeanochi M. et al, 2009	YW	1	0	0	2	1	1	1	0	6
	AE	0	1	1	1	1	1	1	1	
	GD	1	0	1	1	1	1	1	0	
	TW	0	1	1	0	1	1	1	0	
Haddad B. et al, 2015	YW	1	1	1	0	1	0	0	1	6
	AE	0	1	1	1	1	0	0	1	
	GD	1	1	1	1	1	1	1	0	
	TW	1	0	1	2	1	0	0	1	
Pilapil M. et al, 2016	YW	1	1	1	1	1	1	1	1	7
	AE	1	1	1	1	1	1	1	0	
	GD	1	0	0	1	1	1	1	1	
	TW	1	1	1	1	1	0	1	1	
Haddad B. et al, 2018	YW	1	1	1	1	1	1	1	1	8
	AE	1	1	1	1	1	1	1	1	
	GD	2	1	0	1	1	1	0	1	
	TW	2	1	1	1	1	1	1	1	
Berhane Y. et al, 2015	YW	2	0	1	2	1	1	1	0	8
	AE	2	1	1	2	1	0	1	1	
	GD	2	1	1	1	1	1	1	0	
	TW	1	1	1	0	1	1	1	1	
Dessie Y. et al, 2011	YW	2	0	1	2	1	1	1	0	8
	AE	2	1	1	2	1	0	1	1	
	GD	2	1	1	1	1	1	1	0	
	TW	1	1	1	0	1	1	1	1	
Yeshaneh A. et al (2021)	YW	1	0	0	2	1	1	1	1	7
	AE	1	1	1	1	1	1	1	1	
	GD	1	0	1	1	1	1	1	0	
	TW	1	1	1	1	1	1	1	0	
Dereje L. et al (2021)	YW	2	1	1	1	1	1	1	1	8
	AE	2	1	1	1	1	0	1	1	
	GD	2	1	1	1	1	1	1	0	
	TW	2	1	1	0	1	1	1	1	
Biruk. et al (2020)	YW	1	0	0	1	1	1	1	1	7
	AE	1	1	1	1	1	1	1	1	
	GD	1	0	1	1	1	1	1	1	
	TW	1	1	1	0	1	1	1	1	
Rahel. et al (2020)	YW	1	1	0	2	1	1	1	1	8
	AE	2	1	1	1	1	1	1	1	
	GD	1	1	1	1	1	1	1	1	
	TW	1	1	1	1	1	1	1	0	
	YW	1	1	0	2	1	1	1	1	8

Adefala et. al (2020)	AE	2	1	1	1	1	1	1	1	
	GD	1	1	1	1	1	1	1	1	
	TW	1	1	1	1	1	1	1	0	
Tadesse and Gelagay (2019)	YW	1	1	0	2	1	1	1	0	8
	AE	2	1	1	1	1	1	1	1	
	GD	1	1	1	1	1	1	1	1	
	TW	2	1	1	1	1	1	1	0	
Ilesanmi et al (2014)	YW	1	1	0	2	1	1	1	1	7
	AE	1	1	1	1	1	1	1	1	
	GD	1	1	1	1	1	1	1	0	
	TW	0	1	1	0	1	1	1	0	
Aboubacrine et al (2007)	YW	1	1	1	1	0	1	1	1	6
	AE	1	0	1	1	0	0	1	1	
	GD	1	1	1	1	1	1	1	0	
	TW	1	0	1	1	0	0	1	1	
Ajayi et al (2022)	YW	1	1	1	1	0	1	1	1	6
	AE	1	0	1	1	0	0	1	1	
	GD	1	1	1	1	1	1	1	0	
	TW	1	0	1	1	0	0	1	1	
Ankunda et al (2016)	YW	1	1	1	2	1	1	1	1	6
	AE	1	0	1	0	0	1	1	1	
	GD	1	0	1	0	0	1	1	1	
	TW	1	0	1	0	0	1	1	1	
Keetile et al (2018)	YW	1	1	1	2	1	1	1	1	8
	AE	1	1	1	1	1	1	1	1	
	GD	1	1	1	1	1	1	1	0	
	TW	1	1	1	1	1	1	1	1	
Nakiganda et al (2017)	YW	1	1	0	2	1	1	1	1	8
	AE	1	1	1	1	1	1	1	1	
	GD	1	1	1	1	1	1	1	1	
	TW	1	1	1	1	1	1	1	1	
Obi et al (2009)	YW	1	0	1	1	1	0	1	0	6
	AE	1	0	1	1	1	0	1	0	
	GD	1	0	1	1	1	0	1	0	
	TW	1	1	1	2	1	1	1	1	
Ragnarsson et al (2011)	YW	1	0	1	1	1	0	1	0	7
	AE	1	1	1	2	1	1	1	1	
	GD	1	0	1	1	1	0	1	0	
	TW	1	1	1	2	1	1	1	1	
Wagner et al (2010)	YW	1	1	1	1	1	1	1	1	8
	AE	0	1	1	2	1	1	1	1	
	GD	1	1	0	1	1	1	1	1	
	TW	1	1	1	2	1	1	1	1	

Supplementary Table S4: inter-rater agreement for included articles based on the Newcastle-Ottawa Quality Assessment tool, 2023.

Overall Agreement[a]						
		Asymptotic			Asymptotic 95% Confidence Interval	
	Kappa	Standard Error	z	Sig.	Lower Bound	Upper Bound
Overall Agreement	.226	.071	3.181	.001	.222	.231
a. Sample data contains 33 effective subjects and 4 raters.						

Supplementary Table S5: Meta-regression result

Random-effects meta-regression
Method: DerSimonian–Laird

Number of obs = 33
Residual heterogeneity:
tau2 = 0
I2 (%) = 0.00
H2 = 1.00
R-squared (%) = 0.00
Wald chi2(5) = 0.11
Prob > chi2 = 0.9998

_meta_es	Coefficient	Std. err.	z	P>|z|	[95% conf.	interval]
publicationyear	.7523419	3.003652	0.25	0.802	-5.134709	6.639393
samplesize	-.0097575	.0671225	-0.15	0.884	-.1413152	.1218002
Region	-.8804529	14.13384	-0.06	0.950	-28.58227	26.82137
Study_Design	-8.242482	53.0893	-0.16	0.877	-112.2956	95.81064
Gender	-4.963875	29.14495	-0.17	0.865	-62.08692	52.15917
_cons	-1446.204	6045.398	-0.24	0.811	-13294.97	10402.56

Test of residual homogeneity: Q_res = chi2(27) = 2.02 Prob > Q_res = 1.0000
